# Les traumatismes de l’étage antérieur de la base du crane: à propos d'une série de 136 cas

**DOI:** 10.11604/pamj.2015.21.155.3511

**Published:** 2015-06-24

**Authors:** Abdelali Bouchaouch, Fahd Derkaoui Hassani, Hilal Abboud, Jeff Ntalaja Mukengeshay, Nizare El Fatemi, Rachid Gana, Moulay Rchid El Maaqili, Najia El Abbadi, Fouad Bellakhdar

**Affiliations:** 1 Service de Neurochirurgie de l'Hôpital Ibn Sina de Rabat, Maroc

**Keywords:** Traumatismes, étage antérieur, base du crane, trauma, fore floor, base of the skull

## Abstract

Les traumatismes de l’étage antérieur de la base du crâne représentent 15 à 20% des traumatismes crâniens en général. Ils menacent les structures neuro-encéphaliques sus jacentes et sont très souvent responsables de brèches ostéo-méningées exposant au risque infectieux. Notre travail a concerné 136 dossiers exploitables de traumatisme de l’étage antérieur de la base du crâne colligés sur une période de 10 ans entre janvier 2003 et décembre 2012. Le diagnostic a été suspecté devant les signes cliniques évocateurs (ecchymose péri-orbitaire, rhinorrhée…) et a été confirmé dans la plupart des cas par la TDM. Le traitement idéal est la fermeture chirurgicale de la brèche en association aux moyens médicaux (vaccination, anti-épileptiques, mesures de réanimation…) Le moment idéal de la réparation est au-delà de la 72^ème^ heure après la diminution de l'oedème cérébral en cas d'absence d'une lésion intracrânienne nécessitant une intervention en urgence. Notre équipe ne pratiquant pas la voie endoscopique, l'abord frontal est souvent indiqué. Le pronostic dépend des lésions cérébrales associées et surtout de la présence d'une brèche dont le diagnostic et la réparation doivent être les plus rapides et les plus précis possibles. Ainsi toute rhinorrhée post-traumatique nécessite une exploration systématique, le timing idéal: c'est la disparition de l'oedème cérébral pour faciliter l'exploration, ceci est en général possible à partir de la 72^ème^ heure sauf dans les cas associés à une autre lésion intra crânienne nécessitant une exploration en urgence.

## Introduction

Les traumatismes de l’étage antérieur représentent une entité particulière des traumatismes crâniens. Il s'agit du sous type le plus fréquent des traumatismes de la base du crâne et représentent 15 à 20% des traumatismes crâniens en général. Ces traumatismes menacent les structures neuro-encéphaliques sus-jacentes et sont très souvent responsables de brèches ostéoméningées exposant au risque infectieux. Les causes de ces traumatismes sont dominées par les accidents de la voie publique. Le diagnostic des fractures et des brèches ostéoméningées de l’étage antérieur peut être évident devant un tableau clinique typique. Des fois il est délicat et échappe aux investigations radiologiques standards. La TDM a résolu en partie ce problème; cependant certaines brèches durales peuvent passer inaperçues au scanner d'où l'intérêt de l'IRM. La diversité du tableau clinique et des lésions observées au cours des traumatismes de l’étage antérieur justifie l'intérêt d'une prise en charge pluridisciplinaire des patients qui en sont victimes. Enfin, le pronostic de ces traumatismes dépend des lésions cérébrales associées et de la présence d'une brèche ostéo-durale dont le diagnostic doit être le plus rapide et le plus précis possible.

## Méthodes

Notre étude concerne des malades pris en charge pour un traumatisme de l’étage antérieur au service de Neurochirurgie de l'hôpital IBN SINA de Rabat sur une période de 10 ans allant du 1^er^ Janvier 2003 au 31 Décembre 2012. L'analyse a porté sur les éléments épidémiologiques, cliniques, les explorations neuroradiologiques, l'attitude thérapeutique et sur l’évolution.

## Résultats

Nous avons réalisé une étude rétrospective sur un total de 1900 cas de traumatismes crâniens hospitalisés au service de Neurochirurgie de l'hôpital Ibn Sina de Rabat durant la même période. Nous avons retrouvé 136 dossiers exploitables, ce qui représente une fréquence de 7,16%. On a noté une nette prédominance masculine avec 118 hommes (soit 86,76% de l'ensemble) contre 18 femmes, soit 13,24% des cas. Soit un sexe ratio de 6,5H/1F. Dans notre série, l’âge moyen était de 32,7 ans avec des extrêmes de 16 ans à 75 ans. La population jeune dont l’âge est compris entre 16 et 35 ans était la plus concernée et représentait 66%. Les accidents de la voie publique (AVP) représentent la première cause du traumatisme dans notre série avec une fréquence de 69%. Les chutes occupent la seconde place avec une fréquence de 19% contre 12% pour les agressions. La rhinorrhée était présente chez 10,37% des patients en préopératoire et chez un seul patient en post-opératoire chez qui elle était tarie spontanément. 12,3% des malades ont présenté une épistaxis à l'admission, et 3% avaient une otorrhagie. Parmi nos patients, 82% avaient un traumatisme crânien bénin, 16% un traumatisme crânien modéré et 2% un traumatisme crânien grave. Un syndrome méningé a été noté chez 3 patients de notre série, tandis qu'on a relevé un cas de crises convulsives, un cas d'hémiparésie (dû à une pneumocéphalie compressive), et un cas de paralysie faciale dû à une fracture du rocher. Une plaie du scalp a été retrouvée chez 36,8% de nos malades, avec issue de LCR et de matière cérébrale à travers la plaie chez 2 patients. L'ecchymose périorbitaire était présente chez 67% des patients, dans 28% des cas elle était unilatérale et dans 72% des cas elle était bilatérale.

**Figure 1:**
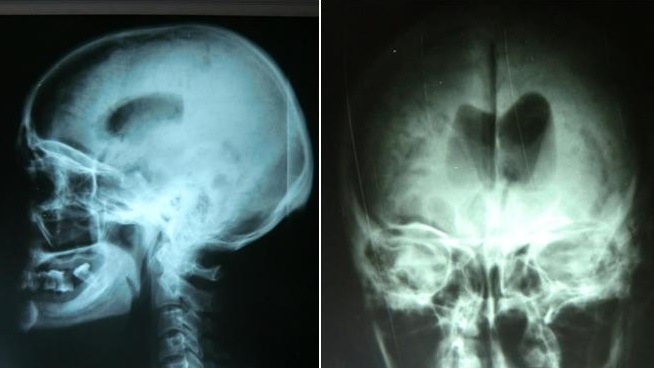
Radiographie du crâne face et profil objectivant une pneumocéphalie avec pneumoventriculie

**Figure 2:**
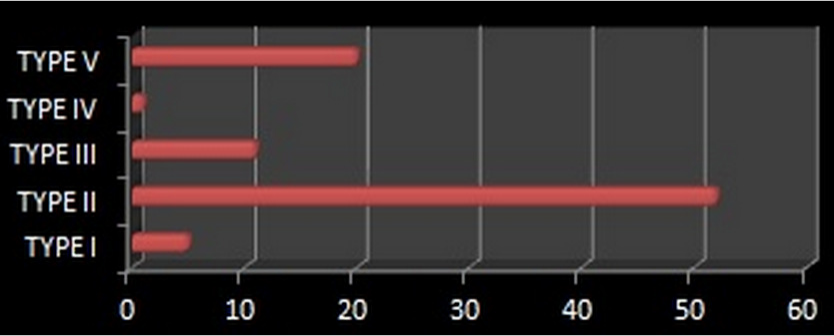
Répartition des malades selon la classification de Fain et Peri

**Figure 3:**
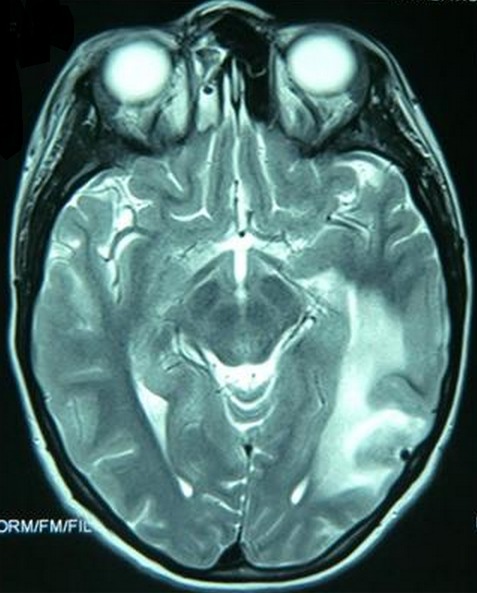
IRM cérébrale objectivant une brèche ethmoïdale droite

La radiographie standard du crâne a été réalisée chez 63% de nos malades, elle a permis d'objectiver des lésions osseuses telles les fractures de la voute, les embarrures, les lésions du sinus frontal, de dépister une pneumocéphalie et chez un cas elle a permis de visualiser une pneumoventriculie (Figure 1). La TDM cérébrale a été réalisée chez tous nos patients, en fenêtre osseuse et parenchymateuse, avec reconstructions coronales. Elle a permis de suspecter une fracture de l’étage antérieur de la base du crâne dans 87,7% des cas, une pneumocéphalie était présente chez 25% des patients. Les lésions osseuses de l’étage antérieur ont été classées selon la classification de Fain et Péri [1] (Figure 2): type I: fracture de la paroi antérieure du sinus frontal; il peut exister de façon exceptionnelle des fractures de la paroi postérieure; type II: enfoncement médiofacial; c'est la dislocation naso-orbitoethmoïdo-frontale ou DONEF de la classification de Paul Tessier, une disjonction craniofaciale de type Le Fort II ou III peut y être associée; type III: fractures de la voûte irradiées à la base avec trait simple ou embarrure; en cas d'embarrure, les lésions de la base sont plus fréquentes de même que les atteintes de la dure-mère; type IV: association des types II et III; type V: lésions exceptionnelles isolées de l’étage antérieur. Dans notre étude, uniquement deux patients ont bénéficié d'une IRM cérébrale dans le cadre du bilan préopératoire pour pouvoir localiser la BOM qui n’était pas évidente au scanner (Figure 3).

**Figure 4:**
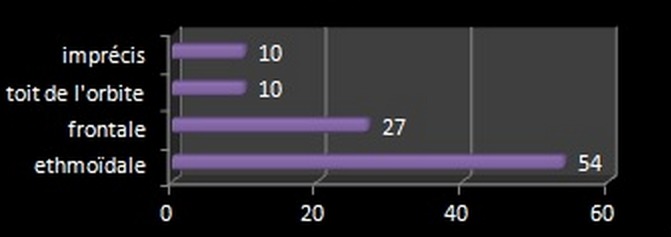
Localisation de la brèche

La prise en charge thérapeutique a toujours commencé par les mesures de réanimation et de mise en condition. Une antibiothérapie préventive a été de mise chez tous les patients de la série qui avaient un traumatisme crânien ouvert. Chez les autres malades, l'antibiothérapie n'a été de mise qu’à visée curative en cas de complications infectieuses neuro-méningées. Dans notre étude, 91 patients ont bénéficié d'une exploration chirurgicale de leur étage antérieur soit 66,91% des cas. Un abord frontal bilatéral a été réalisé chez 67 malades, un abord frontal unilatéral chez 19, une exploration à travers une esquillectomie chez un malade, et une exploration après reprise d'un volet frontotemporal déjà réalisé pour hématome extra dural chez un seul malade. 3 comptes rendus opératoires n'ayant pas été retrouvés Au total 66 explorations extra durales ont été réalisées (75%) contre 22 (25%) d'explorations intra et extra durales. 119 brèches ont été retrouvées chez 80 malades alors que l'exploration s'est révélée infructueuse chez 9% des cas. Le siège de la brèche a été largement dominé par la localisation ethmoïdale chez 54 patients (Figure 4). Parmi nos patients 7 subi une levée d'embarrure avec craniectomie à os perdu, 6 ont bénéficié de l’évacuation d'un hématome extra dural compressif et 3 malades d'un parage avec fermeture d'une plaie cranio-cérébrale.

L’évolution a été jugée favorable chez 95,6% des patients opérés. Aucun cas de mortalité péri-opératoire n'a été noté dans notre série. L'analyse des dossiers de nos malades a permis de constater que 9 patients ont présenté au moins une complication, soit 6,6% des TEA. Parmi ces complications, on a noté 4 cas de brèches per-opératoire soit une fréquence de 4,39%, 2 cas de méningites purulentes traitées par antibiothérapie de 10 jours avec bonne évolution, 2 cas de persistance de rhinorrhée ayant nécessité une reprise chirurgicale soit 2,19%, et un cas de chute du lit ayant occasionné un hématome extradural nécessitant une évacuation chirurgicale avec bonne évolution. Seuls 22 de nos patients ont été revus en consultation selon les informations recueillies sur les dossiers médicaux soit 16,17% des malades, avec un suivi moyen de 17,3 mois avec des extrêmes de 1 à 78 mois.

Les séquelles notées étaient dominées par une hyposmie chez les malades présentant une brèche médiane avec abord frontal bilatéral soit 74% des malades, avec anosmie chez seulement 3 patients ayant nécessité la désinsertion des filets olfactifs. Les autres séquelles notées étaient représentées par 2 cas de céphalées persistantes jugulées par le traitement médical, un cas d’épilepsie secondaire jugulée par le valproate de sodium, un cas d'aphasie, et un cas de parésie faciale.

## Discussion

Les données épidémiologiques de notre série rejoignent celles de la littérature avec une nette prédominance masculine (86,7% d'hommes), un âge moyen proche de la trentaine (32 ans), et une large prédominance des accidents de la voie publique comme facteur étiologique (69% des cas). Lorsque le traumatisme crânien est grâve (2% des cas de notre série), il est difficile de rechercher la rhinorrhée ou l'anosmie, dans ce cas la recherche d'une lésion de l’étage antérieur entre dans le cadre du bilan complet surtout en cas de présence d'ecchymose péri-orbitaire ou d'enfoncement cranio facial.

**Figure 5:**
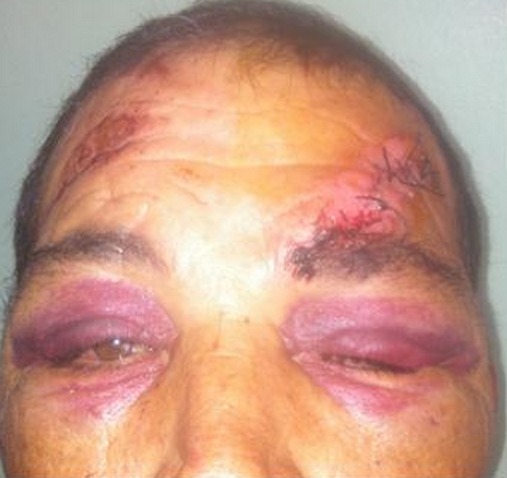
Ecchymose périorbitaire bilatérale associée à une plaie frontale gauche

La majeure partie de nos patients étaient conscients à l'admission (82%) facilitant ainsi la recherche de signes en faveur d'une brèche ostéo-méningée, très comparable à la série de Benbihi [2] (81%). Cependant pour Piek [3], le traumatisme crânien était grave pour 13,51% des cas, modéré pour 50% et léger pour 36,49% des cas. La brèche ostéo-méningée (BOM) post-traumatique correspond à une solution de continuité ostéoméningée qui permet au liquide cérébrospinal (LCS) de s’écouler dans une cavité aérique de la base du crâne. Ainsi la rhinorrhée était présente chez 10,37% de tous les patients hospitalisés pour fracture de l’étage antérieur dans notre service. Elle survient dans la moitié des cas durant les premières 48h avec augmentation de cette fréquence de 8% durant la première semaine [4]. D'expression variable, elle est difficile à mettre en évidence chez un patient intubé, il faut penser à la rechercher le matin sous la forme d'une tache claire sur l'oreiller. Dans sa forme typique, de diagnostic aisé, elle est décrite comme un écoulement par le nez de liquide clair, intermittent, souvent favorisé par la position tête penchée en avant. La recherche de glucose dans cet écoulement par bandelette est définitivement obsolète, du fait de la présence de celui-ci dans les sécrétions nasales. Lorsque le recueil de l’écoulement est possible, c'est le dosage de la b2-transferrine, protéine hautement spécifique du LCS, absente des autres fluides de l'organisme, qui confirme la rhinorrhée [1]. Cette rhinorrhée se complique de méningite dans 7 à 30% [1, 4]. Dans notre série, 3 cas de méningites étaient notés parmi les 14 patients présentant une rhinorrhée franche à l'admission soit une fréquence de 21,4%. Herbella [5] a étudié la relation entre le « raccoon eyes sign » et les fractures de la base du crâne dans une étude faite sur 50 cadavres. Il a trouvé que ce signe était associé aux fractures de la base du crâne dans 48% des cas. Dans notre série, 67% des patients présentaient une ecchymose péri-orbitaire (Figure 5), dans les trois quarts des cas elle était bilatérale. Par conséquent ce signe reste utile pour s'orienter vers une fracture de la base du crâne en particulier de l'os frontal. Une plaie cranio-cérébrale a été associée au traumatisme de la base chez 36,8% de nos malades majorant ainsi le risque infectieux lorsqu'il ya un retard de diagnostic et de prise en charge.

Les radiographies du crâne ne sont plus d'actualité dans le bilan des traumatismes de l’étage antérieur de la base du crâne. Lorsqu'ils sont réalisés, ils peuvent objectiver une fracture de la voute, du sinus frontal ou une pneumocéphalie. La tomodensitometrie est l'examen clé dans le bilan lésionnel des fractures de la base du crâne, des coupes fines de 1 à 2 mm dans les plans coronal et axial seront réalisées en fenêtre osseuse et parenchymateuse. Elle permettra d'objectiver le défect même en l'absence de rhinorrhée [6], son siège, le nombre de fractures, la présence de pneumocéphalie ou d'autres lésions associées (hématome extra ou sous dural, contusion, embarrure, lésions du massif facial…) Elle permet enfin de classer la lésion et de poser l'indication opératoire. Certains auteurs [7] ont proposé une méthode de classification automatique informatisée qui serait plus objective et permettrai un gain de temps considérable. L'IRM n'a pas d'intérêt en urgence vu que le scanner haute résolution permet une bonne analyse des lésions, cependant elle est très utile pour localiser la brèche en présence de rhinorrhée alors que le scanner ne montre pas de brèche. La prise en charge thérapeutique a comme buts de protéger l'encéphale sur les plans mécanique et infectieux, tarir l’écoulement et fermer une éventuelle brèche ostéo-méningée, prévenir les méningites à répétition qui engagent le pronostic vital du malade.

La mise en condition du patient commence sur le lieu du traumatisme surtout en cas de coma avec le maintien des fonctions vitales, l'intubation oro-trachéale (jamais d'intubation naso-trachéale ni de sonde nasogastrique en cas de suspicion d'une fracture de l’étage antérieur [8]). Une vaccination anti-pneumococcique est toujours de mise. L'utilisation d'une antibiothérapie prophylactique est très controversée et n'a pas prouvé son efficacité dans la prévention de méningite en cas de trauma crânien ouvert. Le traitement anti-comitial a été largement utilisé dans notre série surtout en cas de contusion cérébrale, on préfère le valproate de sodium à la dose de 1,5g par jour au phénobarbital qui présente un grand nombre d'effet secondaires. L'acétazolamide a été utilisé chez les patients présentant une rhinorrhée à la dose de 750mg par jour. Les ponctions lombaires déplétives peuvent être d'un grand apport pour favoriser la cicatrisation spontanée d'une BOM post-traumatique. Parfois, on aura recours à un drainage spinal placé pendant quelques jours.

**Figure 6:**
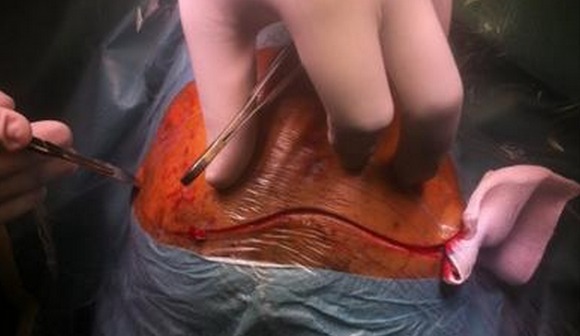
Vue per-opératoire montrant une incision coronale suivant la ligne d'implantation des cheveux

**Figure 7:**
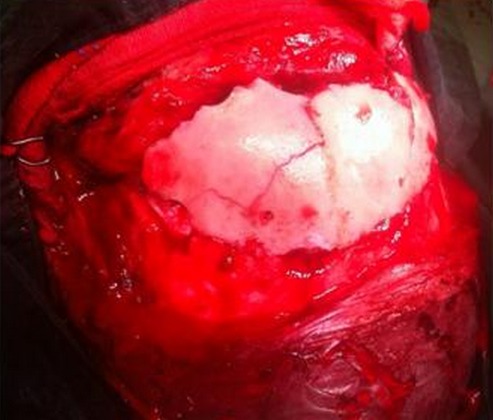
Confection du volet pour une voie transfrontale

Pour explorer un étage antérieur de la base du crâne notre équipe utilise les voies neurochirurgicales classiques qui commencent par une incision coronale allant d'un tragus à l'autre suivant la ligne sinusoïdale d'implantation des cheveux (Figure 6). Le décollement du lambeau de scalp vers l'avant est associé à la découverte des muscles temporaux jusqu'aux piliers orbitaires externes. Il faut prendre garde à ne pas dénuder ces muscles trop bas pour ne pas léser la branche frontale du nerf facial. Par ailleurs, selon leur emplacement et leur importance, les plaies cutanées peuvent être utilisées pour l'exploration voire le traitement des fractures. A ce stade, il y a trois manières d'accéder aux lésions de la base, notamment en fonction de leur étendue: la voie transfrontale (Figure 7): au-dessus des sinus, un volet uni- ou bilatéral, donne accès à la base du crâne en limitant les risques d'anosmie; la voie trans-sinusienne: elle réalise un volet au ras des arcades sourcilières traversant les sinus frontaux. Elle permet un accès plus tangentiel vers la base du crâne et ses lésions, en imposant un écartement moindre au cerveau pour accéder au jugum, ce qui limite le risque d'oedème cérébral. L'anosmie est quasiment inéluctable si l'abord est bilatéral; et la voie transfracturaire ou trans-lésionnelle qui représente souvent un accès associé à l'un des deux précédents. Outre d’éviter parfois la réalisation d'un volet osseux, elle permet l'inventaire des lésions sous jacentes à la fracture.

Quelle que soit la voie d'abord, un temps d'exploration durale est indispensable. Il peut se limiter à un décollement de la dure-mère, très antérieur si les lésions intéressent la face postérieure du sinus frontal, épargnant ainsi l'odorat. Si ces lésions s’étendent en arrière, l'exploration doit pouvoir aller jusqu'au jugum sphénoïdal et au tubercule de la selle sacrifiant ainsi l'odorat. L'exploration intradurale impose l’écartement doux et progressif des lobes frontaux. Si cette voie intradurale est unilatérale, l’épargne olfactive devient possible mais avec le risque d'ignorer une fistule proche des filets olfactifs moins bien explorés. L'abord intradural permet aussi l'accès aux lésions cérébrales éventuelles, mais c'est la voie extradurale qui est la mieux adaptée pour la suture des lésions dure-mériennes. Cette suture se fait par des points simples ou un surjet, à l'aide d'un fil non résorbable 4/0. Pour en garantir l’étanchéité, une doublure doit être assurée par lambeau libre ou pédiculé d’épicrâne garni de colle biologique, ou par les lambeaux aponévrotiques du fascia lata ou du muscle temporal [1, 4, 9]. Il faut proscrire les plasties synthétiques dans cette région à priori contaminée. Au total on a utilisé 74% d'abords frontaux bilatéraux, 21% étaient unilatéraux, chez un cas à travers une esquillectomie, et chez un malade à travers un volet fronto-temporal déjà réalisé pour évacuation d'un hématome extra dural. La brèche a été retrouvée et suturée chez 91% de nos malades alors qu'elle s'est révélée infructueuse chez 9% faisant même discuter l'indication opératoire. Le site le plus fréquent de la brèche est la lame criblée de l'ethmoïde (61,36% de nos patients contre seulement 23% dans la série de Prosser [10]).

La crânialisation des sinus frontaux est la règle après effraction traumatique ou chirurgicale classique de leurs parois, après coagulation et excision la plus complète possible de la muqueuse, la cavité est remplie par des fragments musculaires pour favoriser la fibrose. L’épicrâne est ensuite inversée en dedans pour tapisser la base du crâne fermant ainsi la communication entre la base du crâne et les lobes frontaux, le tout renforcé de colle biologique. Le volet est ensuite remis en place et fixé. L'abord endoscopique endonasal [1, 10] permet de visualiser l’écoulement du LCS au niveau de l'orifice muqueux, soit après localisation de la fistule par les examens d'imagerie, soit lorsque ceux-ci ne la retrouvent pas. Dans le même temps opératoire, elle permet de colmater la brèche au niveau de l’écoulement muqueux. Malheureusement notre équipe n'a pas encore l'expérience de cette voie.

Concernant les indications, pour les fractures du sinus frontal ainsi que les fistules de LCR [8], les indications incluent: une fracture de la table externe avec défomation dysesthétique; une fracture avec obstruction évidente du canal nasofrontal; une fracture de la table interne avec déplacement supérieur à l’épaisseur de la voute prédisant une lacération durale; une fistule de LCS réfractaire; une plaie cranio cérébrale avec fistule de LCS; une pneumocéphalie avec fistule de LCS; la survenue d'une méningite lors de la surveillance d'une rhinorrhée; la survenue tardive d'une rhinorrhée; un défect de la base du crâne avec hernie cérébroméningée à travers ce défect.

Au terme de cette exploration la brèche a été retrouvée et colmatée avec succès chez 91% des malades de notre série avec une bonne évolution clinique. Chez les 9% restants il s'agissait de patients avec un tableau clinique et radiologique évocateur de brèche ostéo-méningée sans rhinorrhée, l'exploration intra et extra durale n'a pas retrouvé de brèche. Une brèche per-opératoire accidentelle est survenue chez 4 patients, mais dans tous les cas la fermeture était aisée. Aucun cas de mortalité péri-opératoire n'a été noté dans notre série. Enfin on a du reprendre 2,19% des patients pour récidive de la rhinorrhée.

Les complications sont essentiellement infectieuses et peuvent se manifester de façon précoce ou tardive. Les séquelles résultent d'une erreur de diagnostic, d'un bilan lésionnel incorrect, d'un traitement primaire mal adapté ou insuffisant ou d'une complication du traitement initial et parfois de lésions graves et complexes. L'anosmie est une séquelle souvent définitive ou de récupération très partielle, elle peut être liée aux lésions initiales quand elles atteignent le complexe frontoethmoïdal ou aux explorations d'une rhinorrhée. 3 cas de notre série ont nécessité une exploration intra et extra durale avec sacrifice du reste des filets olfactifs engendrant ainsi une anosmie définitive, ailleurs il n'y avait pas d'aggravation de l'hyposmie causée par le traumatisme.

## Conclusion

Toute rhinorrhée post-traumatique nécessite une exploration systématique sans passer par les moyens médicaux, le timing idéal: c'est la disparition de l'oedème cérébral pour faciliter l'exploration, ceci est en général possible à partir de la 72^ème^ heure sauf dans les cas associés à une plaie crânio-cérébrale ou une autre lésion intra crânienne (hématome extra-dural, sous-dural…) nécessitant une exploration en urgence.
